# Cellular Lipids—Hijacked Victims of Viruses

**DOI:** 10.3390/v14091896

**Published:** 2022-08-27

**Authors:** Bozena Omasta, Jana Tomaskova

**Affiliations:** Biomedical Research Center, Institute of Virology, Slovak Academy of Sciences, Dúbravská cesta 9, 845 05 Bratislava, Slovakia

**Keywords:** lipids, virus, virus–host interaction, cholesterol, metabolism

## Abstract

Over the millions of years-long co-evolution with their hosts, viruses have evolved plenty of mechanisms through which they are able to escape cellular anti-viral defenses and utilize cellular pathways and organelles for replication and production of infectious virions. In recent years, it has become clear that lipids play an important role during viral replication. Viruses use cellular lipids in a variety of ways throughout their life cycle. They not only physically interact with cellular membranes but also alter cellular lipid metabolic pathways and lipid composition to create an optimal replication environment. This review focuses on examples of how different viruses exploit cellular lipids in different cellular compartments during their life cycles.

## 1. Introduction

Viruses are classified as obligate intracellular parasites, which means they must complete their life cycle in live host cells. Upon entry into the cell, they use various mechanisms to rewire metabolic pathways and produce progeny. Over the past few decades, the role of lipids during viral replication has been intensively studied, and cellular lipids have been shown to play a crucial role in viral replication (reviewed in [1]). Since lipids are the main constituents of the plasma membrane, they represent the first barrier to the entry of viral particles into the cell. In some cases, lipids serve as viral receptors [2,3,4] or co-receptors [5]. During genome replication and virion assembly, some viruses induce remodeling of cellular organelles [6,7,8] and use them as sites of replication [9] or protein storage [10]. Posttranslational lipid modifications of viral proteins are frequently used during the assembly of newly synthesized virions [11]. Moreover, lipids represent a great source of energy needed for the completion of the viral life cycle, which is an energy-demanding process [12]. Viruses use various cellular organelles to facilitate each life cycle step to achieve successful replication. In this review, we summarize how different viruses utilize cellular lipids from distinct cellular compartments to complete their life cycle.

## 2. The Roles of Plasma Membrane Lipids in Virus Entry

The plasma membrane, prevailingly made up of phospholipids, separates the intracellular environment from the extracellular and represents the first barrier for viral entry into the host cell. Most viruses overcome this barrier by binding to cellular receptors located within the plasma membrane. Membranous lipids facilitate virus entry by serving as viral receptors or as main constituents of platforms for gathering viral receptors. Receptors can be constitutively expressed in these platforms or translocated to them upon virus binding.

**Figure 1:**
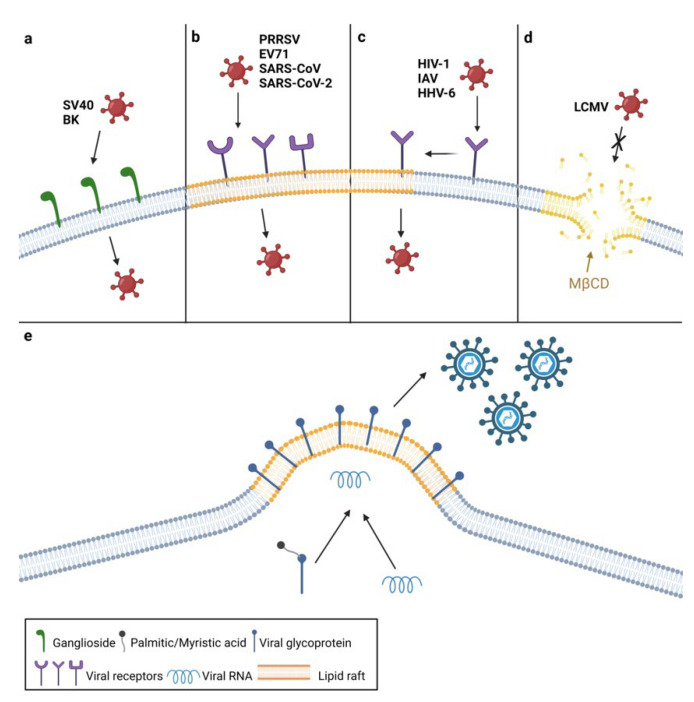
Roles of lipids during viral entry. (**a**) Lipids as viral receptors. Viruses such as polyomaviruses can interact with gangliosides on the cell surface and utilize them for entry into the cell. (**b**) Lipid rafts as gathering sites for viral receptors. Some viruses bind to receptors, which are located at the lipid rafts. (**c**) Viral receptors are translocated to the lipid rafts after virus binding. Some viruses bind to non-raft receptors and induce their translocation to lipid rafts, which facilitate internalization. (**d**) Role of non-raft lipids in viral entry. Arenaviruses require non-raft cholesterol in the plasma membrane for entry into the cell. Sequestering of cholesterol using MβCD enables arenaviruses to enter the cell. (**e**) Roles of lipids during viral assembly and budding. Some viral proteins require myristoylation or palmitoylation (black) to target the lipid membrane, into which they are incorporated. Assembly of newly synthesized virions of some viruses occurs through lipid rafts. Created with BioRender.com.

One example of viruses that bind directly to plasma membrane lipids to initiate infection are polyomaviruses (Figure 1a). They can enter the cell by binding to the plasma membrane’s gangliosides, which are glycosphingolipids linked with one or more sialic acids. Particularly, simian virus 40 (SV40) utilizes GM1 ganglioside, while the BK virus enters the cell by binding to gangliosides GD1b and GT1b [2,13]. Rotaviruses also bind to GM1, and their internalization is cholesterol-dependent [14]. 

Receptors of some viruses are located in unique, detergent-insoluble membrane areas rich in cholesterol, sphingolipids, and GPI-anchored proteins termed lipid rafts. Lipid rafts make up approximately 15 to 20% of the total plasma membrane area and play an important role in various processes such as membrane signaling and signal transduction [15,16]. Since these lipid microdomains are less fluid than the rest of the membrane, they often serve as platforms to concentrate components needed for membrane fusion and endocytosis such as clathrin and caveolin [17]. This type of viral entry is known as raft-dependent and is used by both RNA and DNA viruses (extensively reviewed in [18]).

Some cellular receptors and co-receptors are constitutively expressed in lipid rafts (Figure 1b). Porcine reproductive and respiratory syndrome virus (PRRSV) glycoproteins Gp3 and Gp4 were found in association with lipid rafts during entry. Further experiments confirmed that CD136, the PRRSV receptor, is raft-located and that raft disruption leads to impaired PRRSV entry and subsequent virus titer reduction [19]. The VP1 protein of Enterovirus 71 (EV71) binds to the SCARB2 receptor, which is also located in lipid rafts. Cholesterol depletion inhibits viral propagation in EV71-infected cells in a dose-dependent manner, whereas its replenishment restores the EV71 viral titer [20]. Similarly, severe acute respiratory syndrome coronavirus (SARS-CoV) enters the cell by binding to angiotensin-converting enzyme 2 (ACE2), which is constitutively associated with lipid rafts. During the entry, ectodomain S1188HA of SARS-CoV S protein interacts with cellular lipid rafts. The depletion of cholesterol did not affect the binding of S protein to ACE2, indicating that raft cholesterol is required for the concentration of ACE2 receptors, thus enabling SARS-CoV’s internalization [21]. However, it remains unknown whether this is the case with all types of cells, since results obtained by another group of researchers showed that ACE2 did not colocalize with lipid rafts in Chinese hamster ovary (CHO) cells [22]. Recent findings show that the entry of SARS-CoV-2, the agent causing the ongoing pandemic, is also dependent on lipid rafts [23]. Since lipid rafts serve as a platform for the concentration of ACE2 receptors, SARS-CoV-2’s entry is negatively affected by cholesterol depletion and subsequent raft disruption [24]. 

In other cases, cellular receptors and co-receptors are not expressed in the lipid rafts constitutively but are translocated to them upon virus binding to the cell surface (Figure 1c). One example is the human immunodeficiency virus 1 (HIV-1), which enters the host cell by binding its viral envelope glycoprotein gp120 to the CD4 receptor and CXCR4 or CCR5 co-receptors [25,26]. Mañes and colleagues showed that the binding of gp120 to CD4 causes a lateral reorganization of rafts, bringing the complex into proximity of the rafts containing HIV co-receptors, which leads to the formation of a trimolecular entry complex [27]. HIV-1 is unable to enter the host cell in the presence of methyl-β-cyclodextrin (MβCD), which depletes membrane cholesterol and disperses lipid rafts [28], confirming the importance of lipid rafts for HIV-1 entry [29]. The disruption of raft microdomains by cholesterol depletion inhibits the efficient entry of several herpesviruses, including the human herpesvirus 6 (HHV-6) [30], the herpes simplex virus 1 (HSV-1) [31], and the varicella-zoster virus (VZV) [32,33]. HHV6’s attachment to the cell surface triggers the translocation of its cellular receptor, CD46, into lipid rafts. Moreover, the HHV-6 glycoproteins gB and gQ1 are also associated with lipid rafts immediately after infection, with the latter being more abundant, as it is a part of the glycoprotein complex that binds CD46 [30]. However, relocation of HSV-1 receptor nectin-1 into rafts is not induced by HSV-1’s attachment to the cell surface but by the presence of αVβ3-integrin at the plasma membrane, which enables HSV-1 to enter the cell via a route dependent on lipid rafts and to continue internalization through acidic endosomes [34]. The gB glycoprotein of HSV-1, which is conserved among all herpesviruses, interacts with lipid rafts during the entry process [31]. Since gB is thought to have a fusogenic function, it is reasonable to assume that cholesterol plays a role in the fusion of viral and cellular membranes [35]. 

Another manner of raft involvement in the entry process represents the binding of SV40 to the major histocompatibility complex I (MHCI) that triggers the relocalization of viral particles into lipid rafts, where they bind to GM1 and induce entry through caveolae, a special type of lipid raft enriched in sphingolipids and cholesterol stabilized by cholesterol-binding caveolins [36,37]. The coxsackie B virus also uses caveolae-mediated endocytosis for internalization; however, the coxsackie B virus and adenovirus receptor (CAR) is not located in caveolin-containing caveolae but in specialized lipid rafts, known as tight junctions and adherens junctions [38]. 

Lipid rafts are also crucial for the entry of the influenza A virus (IAV) since it enters the cell via raft-dependent endocytosis, and its receptor-binding glycoprotein hemagglutinin (HA) was found to interact with lipid rafts during entry into the cell [39,40]. The attachment of IAV HA to its receptor induces clustering of lipid rafts and tyrosine kinases, leading to their activation and subsequent internalization of IAV mediated by the PI3K/Akt signaling pathway [41].

Some viruses require the presence of non-raft lipids during their entry (Figure 1d). One of the best examples is the lymphocytic choriomeningitis virus (LCMV), which enters the host cell by utilizing the widely expressed cell surface receptor—α-dystroglycan (α-DG) [42]. Viral titers were significantly lower in cells treated with MβCD before infection, indicating that cholesterol plays an important role in the LCMV life cycle. Further experiments showed that α-DG is not associated with lipid rafts but with non-raft cholesterol, thus, sequestering of cholesterol affects the internalization of LCMV particles [43]. 

Another example is the hepatitis C virus (HCV) that circulates in the bloodstream in complex with apolipoproteins and requires low-density lipoprotein receptors (LDL-R) in addition to other high-affinity receptors for entry into target cells. It seems that interaction with LDL-R brings the virus into the vicinity of other entry co-factors [44,45].

All the aforementioned examples show that viruses can enter a host cell using a variety of mechanisms, but plasma membrane lipids are crucial to each and every one of them.

## 3. The Roles of Plasma Membrane Lipids in Virus Assembly and Egress

In addition to the initial stages of the viral life cycle, lipid rafts often play an important role during virion assembly and budding (Figure 1e). For example, it has been shown that lipid raft cholesterol plays a critical role in viral protein–protein interactions during the synthesis of infectious virions whose assembly takes place in raft microdomains. This phenomenon occurs during the infection with Newcastle disease virus (NDV), an avian paramyxovirus. It has been shown that structural NDV proteins associate with lipid rafts, suggesting their involvement during virion assembly and budding. This was confirmed by sequestering cholesterol from the plasma membrane, which resulted in the production of structurally abnormal virions with reduced infectivity [46]. Newly synthesized particles were able to attach to the plasma membrane but unable to fuse with it. Additionally, Laliberte and colleagues demonstrated that the observed virion defectiveness occurs because lipid rafts are required for protein interactions between NDV HN and F proteins during the assembly of nascent virions. Loss of HN and F interaction prevents the activation of F protein, responsible for the initiation of virus–host membrane fusion [47]. Lipid microdomains serve as assembly sites for other paramyxoviruses as well, such as respiratory syncytial virus [48], measles virus [49], and Sendai virus [50]. 

IAV virion assembly also takes place in lipid rafts since HA, an IAV surface protein, was found in association with these lipid microdomains during the budding of nascent virions [40]. In addition, palmitoylation of HA protein was shown to be crucial for its targeting to the membrane during assembly and incorporation into newly synthesized IAV virions [51,52]. 

Similarly, the HIV Gag protein is associated with lipid rafts during virion budding [53], and as in the case of IAV, the Gag protein requires posttranslational addition of another fatty acid—myristate—for successful targeting to lipid rafts [54]. As was previously stated, lipid rafts are rich in cholesterol, so it is not surprising that HIV enhances cholesterol biosynthesis, which is then redirected to lipid rafts [55]. Furthermore, lipid signaling is crucial for the translocation of HIV structural proteins to raft microdomains and subsequent virion assembly [56,57]. Additionally, HIV is capable of infecting other target cells by direct cell-to-cell transmission, also known as virological synapses, without leaving the infected cell [58]. Jolly and Sattentau suggested that this type of transmission requires lipid rafts, as the treatment with MβCD prevented the formation of virological synapses between T cells [59]. 

## 4. Endosomal Membrane Lipids Facilitate Virion Delivery

There are a few ways through which viruses are internalized into the cell. Although some enveloped viruses enter the cells by fusing with the cytoplasmic membrane, most non-enveloped and enveloped viruses enter the cell via endocytosis. After receptor binding, the cytoplasmic membrane encloses the virus, thus creating an endocytic vesicle that enters the cytoplasm. The endocytic vesicle fuses with an early endosome that later matures into a late endosome which differs in lipid and protein composition, as well as pH level [60]. To start replicating, viral RNA needs to be released from endosomes. The release can occur through the lowering of the pH in the endosome leading to conformational changes in viral proteins, causing membrane fusion and subsequent release of the viral genome into the cytoplasm. Apart from pH lowering, the lipid composition of the endosomal membrane also plays an important role in membrane fusion, as it is crucial for deciding the endosomal compartment in which the virus will fuse [60]. 

Upon entering the endosome, Semliki Forest virus (SFV) glycoprotein E1 undergoes conformational changes that are dependent on low pH and result in the formation of E1 homotrimer and exposition of the fusion protein, crucial for the interaction of the viral and endosomal membrane. It has been demonstrated that E1 is preferably inserted in areas rich in cholesterol and sphingolipids and that it interacts with cholesterol during membrane fusion [61,62]. Another alphavirus, the Chikungunya virus interacts with cholesterol during membrane fusion, as its fusion rate is dependent on the concentration of membranous cholesterol [63]. Similarly, the presence of cholesterol in the endosomal membrane is crucial for membrane fusion of the West Nile virus, a member of the *Flaviviridae* family [64].

Kobayashi and Hirabayashi discovered lipid rafts rich in a phospholipid known as lysobisphosphatidic acid (LBPA), termed LBPA microdomains, found in the endocytic pathway, particularly in the inner leaflet of late endosomes [65]. Later, it was discovered that apart from its involvement in cholesterol homeostasis and sphingolipid metabolism, LBPA often plays a role during viral infection [66]. Some viruses, such as dengue virus (DENV) and vesicular stomatitis virus (VSV), use LBPA as a cofactor of membrane fusion, since pH lowering alone is not enough to cause changes in protein conformation [67,68]. Moreover, treatment of DENV-infected cells with anti-LBPA monoclonal antibodies was shown to reduce infection [69]. Treatment of cells with anti-LBPA antibodies reduces viral titer of mammarenaviruses Lassa virus (LASV) and LCMV, suggesting that the presence of LBPA in late endosomes is required for the release of nucleocapsid during infection with these viruses [70]. A recent study by Markosyan and colleagues confirmed this hypothesis in the case of LASV by revealing that fusion of LASV with the endosomal membrane and subsequent release of its genetic material begins in early endosomes but is completed in late endosomes enriched in LBPA [71]. In addition, recent studies have shown that LBPA is important for the life cycle of newly discovered SARS-CoV-2 [72,73]. However, more studies are required to understand the exact mechanism behind these findings. 

## 5. Viruses Remodel Endoplasmic Reticulum Membranes

After internalization, viruses that replicate in the cytoplasm begin replicating their genome using various cellular compartments, such as the endoplasmic reticulum (ER), Golgi apparatus (GA), and lipid droplets (LDs), as the sites of replication. These areas are known as replication organelles and usually comprise the viral genome, viral RNA polymerases, and non-structural proteins required for replication. One of the most frequently used organelles is the ER, whose membranes get rearranged to create a safe environment, unapproachable for endonucleases and the host immune system [74]. Moreover, membrane rearrangement provides physical support for gathering cellular and viral parts needed for viral genome replication and virion assembly. Replication organelles can be formed either by invagination or protrusion of the ER membranes. The reorganization of cellular endomembranes often leads to changes in lipid metabolism due to a higher lipid demand [75].

**Figure 2:**
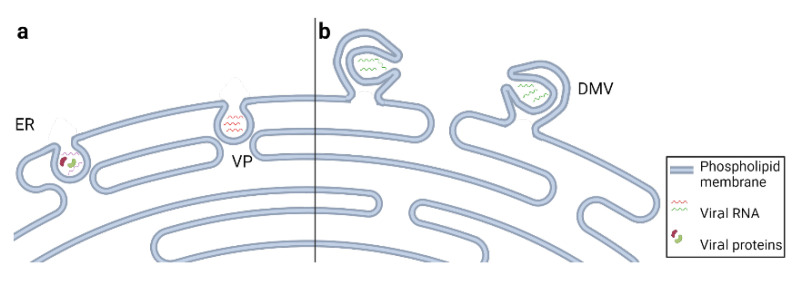
Remodeling of the endoplasmic reticulum (ER) membrane during viral infection. (**a**) Viruses such as Dengue (DENV) and Tick-borne encephalitis virus (TBEV) induce invaginations in the ER known as vesicle packets (VPs). VPs serve as sites for viral genome replication in the case of DENV and as replication and virion assembly in the case of TBEV. (**b**) Genome replication of some coronaviruses and HCV occurs in virus-induced modification of the ER membranes’ double membrane vesicles (DMVs). Both VPs and DMVs are used to produce new virions and protect their genomes from cellular nucleases. Created with BioRender.com.

This phenomenon is characteristic of infection with positive-sense RNA viruses and best described in the case of viruses belonging to the *Flaviviridae* family. One of the examples comprises flaviviruses DENV and Zika virus that induce invaginations of the rough ER, which form invaginated vesicles known as vesicle packets (VPs) [76] (Figure 2a). VPs are single-membrane invaginations into the ER connected to the cytosol via tiny pores, through which required viral and cellular components are exchanged [77]. Although they represent the site of viral genome replication, Cerikan and colleagues have shown that VP formation in DENV-infected cells is not dependent on RNA replication but rather on the expression of NS1-5 polyprotein [78]. In addition to single-membrane invagination VPs, DENV infection induces the formation of more convoluted replication organelles, termed double-membrane vesicles (DMVs). While VPs can be observed in both DENV-infected human and mosquito cells, DMVs are present only in human cells, suggesting that the formation of replication organelles during DENV infection is cell type-specific [79]. DMVs do not contain viral RNA. 

Similar structures were observed in cells infected with tick-borne encephalitis virus (TBEV), another member of the *Flaviviridae* family [80]. While DENV uses VPs only as a site of genome replication, TBEV utilizes VPs for both genome replication and virion assembly [81].

Unlike other flaviviruses, replication of HCV causes the protrusion of the ER and creates organelle-like structures made of DMVs (Figure 2b), which function as the replication sites for HCV [9,82]. These changes are induced by the viral replicase complex, composed of non-structural proteins which assist in viral RNA replication [83]. HCV-induced changes in the ER membrane provide a scaffold for viral genome replication, as well as a safe environment for viral genome due to the lack of proteases and nucleases inside DMVs [84,85]. Moreover, studies showed that when compared to the ER membrane, DMVs comprise a much higher amount of cholesterol, which provides the stability of these vesicles [86]. However, the exact mechanism by which HCV modifies the ER membrane composition remains unknown. In addition, Yu and colleagues showed that HCV non-structural protein NS4B requires posttranslational palmitoylation to form a replication complex [87].

Another group of positive-strand viruses that induce the formation of DMVs is coronaviruses. Their replication–transcription complex (RTC), consisting of non-structural proteins, induces the rearrangement of ER membranes and initiates the formation of DMVs where RTCs then concentrate [88]. In addition, it has been observed that replication of another coronavirus—murine hepatitis virus—occurs in DMVs [89]. Similarly, DMVs were observed in cells infected with the Middle East Respiratory Syndrome coronavirus, which is also a member of the *Coronaviridae* family [90].

Flaviviruses and coronaviruses also induce changes in the smooth ER, which then rearranges and forms into clumps known as convoluted membranes (CMs) [76,91]. In flavivirus- and coronavirus-infected cells, CMs connect with VPs and DMVs, respectively, and make up a membranous web used for the replication of these viruses [77,92]. Welsch and colleagues showed that VPs serve as sites of genome replication [76], while the function of CMs is still not completely understood. However, Ulasli and colleagues proposed that CMs in coronavirus-infected cells are formed due to the aggregation of viral proteins that were not incorporated into virions [93].

## 6. Viruses Utilize Lipids Stored in Lipid Droplets

**Figure 3:**
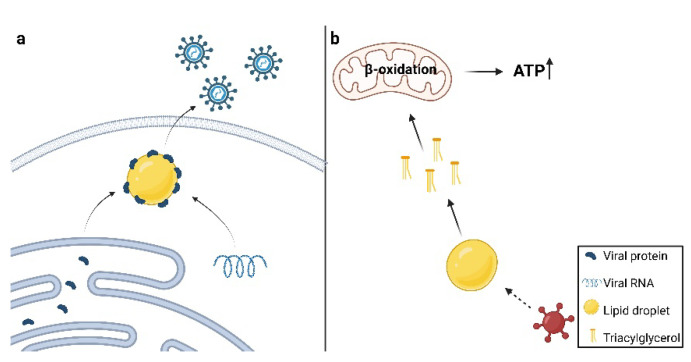
Roles of lipid droplets in viral replication. (**a**) Lipid droplets (LDs) serve as storage for viral proteins. After their synthesis in the endoplasmic reticulum, viral proteins are gathered at LDs. (**b**) LDs serve as a source of energy during viral replication. Some viruses use lipids stored in LDs for energy production. Viral infection induces the release of triglycerides (TAGs) from LDs, which are transported into the mitochondria, where they undergo lipolysis known as β-oxidation and produce large amounts of ATP needed for viral replication. Created with BioRender.com.

After genome replication and synthesis of viral proteins, mature virions are formed by assembling the proteins into viral capsids, inside which the replicated genome is then packaged. As in the case of viral genome replication, lipid structures also play a role during virion assembly. Noteworthy structures during the assembly of the *Flaviviridae* family members are lipid droplets (LDs). LDs are cellular organelles whose core is composed of neutral lipids such as triacylglycerols (TAG) and steryl esters (SE), surrounded by a phospholipid monolayer and structural proteins, which separate them from hydrophilic cytosol [94]. LDs are derived from the endoplasmic reticulum and are primarily used as an energy source. However, they are also known to be involved in cellular processes such as lipid homeostasis, membrane trafficking, and signal transduction [95]. During viral infection, LDs are often used as assembly sites for nascent virions [96] (Figure 3a).

It has been demonstrated that cells infected with HCV and DENV comprise a higher percentage of LDs, which are located close to virus-induced replication sites and used as storage sites for viral proteins. After their synthesis in the ER, the transfer of HCV proteins to the surface of LDs is assured by the creation of membrane bridges between ER and LDs during LD biogenesis [97,98]. The first translocated protein is the HCV core protein, which interacts with diacylglycerol acyltransferase 1 (DGAT1), an enzyme catalyzing the synthesis of TAGs, thus ensuring the incorporation of the core protein into LDs during their biogenesis [10]. Accordingly, it has been observed that inhibition of DGAT1 activity during HCV infection significantly reduces the production of infectious virions [99]. The attachment of the core protein is followed by translocation of nonstructural protein NS5A to the LDs [100], while NS3 and NS4B remain in the ER [101]. Moreover, the replication complexes in the ER are brought to regions associated with LDs, which allows for the initiation of virion assembly [102]. Both core protein and NS4B require palmitoylation during the synthesis of HCV particles [87,103]. HCV uses lipid droplets as sites of virion assembly while activating the production of more lipids via sterol-regulated element-binding protein (SREBP), a transcription factor responsible for the transcription of lipogenic enzymes [104]. On the other hand, DENV uses LDs as a source of lipids instead of de novo synthesis via SREBP (reviewed in [7]). 

LDs also serve as a source of TAGs for the synthesis of very low-density lipoprotein (VLDL) [105,106]. Several studies have confirmed that VLDLs are essential for the secretion and production of infectious HCV virions, as targeting with apolipoprotein antibodies resulted in a decrease in infectious HCV virions [107,108]. Moreover, cells depleted of certain apolipoproteins are not capable of producing infectious HCV virions [109].

In addition to flaviviruses, LDs play a crucial role in the assembly of rotaviruses (RVs), whose replication takes place in discrete cytoplasmic inclusion bodies, termed viroplasms, which are found in association with LDs during RV infection. Apart from non-structural and structural RV proteins, viroplasms were found to contain LD components such as perilipin A and adipophilin. Colocalization of RV NSP5 protein, found in viroplasms with perilipins PLIN1 and PLIN2, markers of lipid droplets, confirmed the association between these two structures [110]. A recent study by Criglar and colleagues has shown that the synthesis of viroplasms and lipid droplets occurs simultaneously, and that disruption of LD homeostasis or inhibition of LD formation has a negative impact on RV virion production. Furthermore, a lipidome analysis showed that the total lipid content of RV-infected cells is considerably higher when compared to mock-infected cells, thus confirming the lipid dependence during RV propagation [111].

Recent findings show that newly emerged coronavirus SARS-CoV-2 utilizes LDs during virion assembly. The SARS-CoV-2 infection causes an upregulation in lipid metabolism and an increase in LD levels. Moreover, treatment of SARS-CoV-2-infected cells with A922500, a DGAT1 inhibitor, significantly reduced the production of infectious virions in a dose-dependent manner. The involvement of LDs in SARS-CoV-2 replication was confirmed by the detection of viral dsRNA in proximity to BODIPY-labeled LDs [112]. Ricciardi and colleagues have demonstrated that the NSP6 protein is responsible for the interaction of SARS-CoV-2 replication areas with LDs [113].

In addition to the storage of viral proteins, lipid droplets are occasionally used as a source of energy (Figure 3b). When exogenous fatty acids are unavailable, some viruses induce the liberation of free fatty acids (FFAs) from TAGs, a major part of LDs. Released fatty acids are transported into mitochondria where they undergo lipolysis known as β-oxidation and generate ATP. This process is known as lipophagy, selective autophagy targeting LDs. During viral infection, cells use autophagy as an anti-viral defense, however, some viruses have found a way to utilize it for their replication [114]. 

Heaton and Randall have demonstrated that DENV uses autophagy to regulate lipid metabolism during its replication [12]. DENV infection induces the formation of autophagosomes, which were observed to colocalize with LDs. Over the course of infection, the size of LDs in DENV-infected cells decreased; on the other hand, inhibition of autophagy caused an increase in the LD area. Moreover, analysis of individual lipid classes showed that TAG levels decreased significantly in DENV-infected cells when compared to mock-infected cells. DENV-infected cells are also characterized by an increased rate of β-oxidation, which was confirmed to be necessary for DENV replication since treatment with Etomoxir led to the reduction in viral titer [12]. In a later study, Zhang and colleagues revealed that DENV-induced lipophagy requires the presence of ancient ubiquitous protein 1 (AUP1), a membrane protein associated with ER and LDs, whose distribution is altered upon DENV infection. Consistently, the production of infectious DENV virions decreased in cells lacking AUP1. NS4A and NS4B proteins were found to initiate AUP1-mediated lipophagy by binding to LDs, causing their translocation to autophagosomes. Although the exact mechanism through which NS4A interacts with AUP1 is not known, it was demonstrated that NS4A interacts with the deubiquitinated form of AUP1 only [115]. 

Lipophagy was also observed during infection with PRRSV. The study by Wang and colleagues proposes that PRRSV induces autophagy through N-myc downstream-regulated gene 1 (NDRG1), which is thought to regulate autophagy through various signaling pathways [116]. PRRSV infection caused downregulation of NDRG1; on the other hand, NDRG1 overexpression inhibited PRRSV replication. Moreover, PRRSV replication increased while cellular LDs decreased in NDRG1-deficient cells. These cells also contained more FFAs, suggesting that NDRG1 plays a role in regulating lipophagy. Inhibition of autophagy by 3-methyladenine caused a reduction in FFAs and increased LD levels, confirming that lipophagy during PRRSV infection is regulated via NDRG1. However, the exact mechanism through which PRRSV interacts with NDRG1 remains to be elucidated [117].

## 7. Conclusions

Viruses have evolved mechanisms through which they are able to exploit host cells. With the increasing availability of tools, we have managed to broaden our understanding of virus–host interactions. Over the last two decades, virus interactions with cellular lipids have been intensively studied. Numerous studies have shown that viruses exploit lipids by altering lipid metabolism, signaling pathways that regulate lipid metabolism, or by remodeling lipid membranes. Even though almost all viruses utilize lipids in similar ways, every family also uses unique mechanisms, and therefore needs to be studied individually. It is not uncommon that one virus can use multiple mechanisms to exploit cellular lipids in the same cell, making their study more complicated. Moreover, the strategy of using cellular lipids and virus-induced changes in lipid metabolism may differ depending on viral strain and cell line, but the reasons for these differences are usually not clear. Although much more research is needed to fully understand virus–lipid interactions, current studies have already shown the importance of cellular lipids in the life cycle of many viruses. Detailed understanding of the viral requirements for cellular lipids may not only improve our knowledge of the virus–host interaction but may also provide new treatment options and opportunities to repurpose existing FDA-approved drugs targeting lipid metabolism as potential antiviral therapeutics. For example, as many viruses require active fatty acid biosynthesis, treatment with lipid-lowering drugs such as statins may have a beneficial effect on reducing viral infections. The advantage of such drugs is that they may be less prone to the development of viral drug resistance because they target host proteins. Therefore, an appealing outcome of research in this field is the prospect of broad-spectrum antiviral therapeutics that target lipid metabolism.
